# Realizing real-time optical molecular imaging in peripheral nerve tissue via Rhodamine B

**DOI:** 10.3389/fmed.2024.1461520

**Published:** 2024-11-26

**Authors:** Jinzheng Wei, Xinyu Guo, Yixi Wang, Yunmeng Zhang, Wei Zhao, Shufeng Han, Chao Liu, Xiaofeng Yang, Wenkai Liang

**Affiliations:** ^1^Department of Orthopaedics, First Hospital of Shanxi Medical University, Taiyuan, China; ^2^Biomedical Engineering Research Center, Shanxi Medical University, Taiyuan, China; ^3^Department of Urology, First Hospital of Shanxi Medical University, Taiyuan, China; ^4^School of Electronic Engineering, Xidian University, Xi’an, China

**Keywords:** OMI technology, peripheral nerve, Rhodamine B, real-time fluorescence imaging, SBR

## Abstract

**Background:**

Iatrogenic nerve injury is a consequential complication during surgery. Thus, real-time imaging of peripheral nerve (PN) possesses significant clinical implications. In recent years, the rapid advancements in optical molecular imaging (OMI) technology have provided essential technical foundations for the implementation of PN fluorescence imaging. This study aimed to realize real-time OMI of PNs via Rhodamine B.

**Methods:**

Phosphate buffered saline (PBS), normal saline (NS), 5% glucose solution (GS), and fetal bovine serum (FBS) were selected for measuring the fluorescence spectra of Rhodamine B solutions prepared in each formulation. Rhodamine B solutions, with varying doses dissolved in 100 μL of each formulation, were prepared and applied to the exposed PNs of the mice for incubation later. To ascertain the optimal formulation and dose of Rhodamine B, an analysis was performed on the signal-to-background ratio (SBR) of the nerves. Based on the experimental results, we proceeded to incubate Rhodamine B solution on the PN tissue of mice and human subjects, as well as on neuronal cells, to verify the binding sites of Rhodamine B with nerve. Subsequently, histological studies were conducted to validate the binding site between Rhodamine B and the nerves. Finally, we injected the optimal combination of Rhodamine B solution into mice via the tail vein and collected the SBR of mouse nerve tissues at different time intervals to determine the optimal pre-injection time. Fluorescence images of various tissues were collected, and Hematoxylin and Eosin (H&E) staining results were observed to determine the metabolism of Rhodamine B in mice and its toxicity.

**Results:**

The excitation peak of Rhodamine B in PBS, NS, 5% GS, and FBS formulations was 554 nm, and the emission peak was 576 nm. In PBS group, the maximum SBR was 15.37 ± 0.68 while the dose of Rhodamine B was 8 nmol. Through *ex-vivo* validation on fresh human nerve tissue and verification using mouse and human tissue sections, we observed fluorescent signals of Rhodamine Bin the regions of nerve tissue and the fluorescence signals were all concentrated on the neuronal cell membranes. After injection, the fluorescent signal in nerve tissue reached its peak at 24 hours (h), coinciding with the highest SBR (5.93 ± 0.92) in mouse nerve tissues at this time point. Additionally, the fluorescence signal could be maintained for at least 48 h. Within 24 h, lung dilation and fusion of alveoli occurred. Then these pathological manifestations gradually diminished, returning to normal at 2 weeks (w), with no significant acute or chronic adverse reactions observed in other tissues.

**Conclusion:**

Rhodamine B enables fluorescence imaging of PNs and has the potential for clinical translation.

## 1 Introduction

Iatrogenic nerve injury is a highly concerning complication in surgical procedures (1). This is primarily attributed to surgeons’ limited ability to visualize nerves clearly during surgery, leading to potential inadvertent nerve cutting, stretching, ligation, piercing, torsion, or thermal damage from electrocautery or hardened bone cement (2). Intraoperative iatrogenic nerve injury can cause irreversible harm to patients, including pain, abdominal distension, limb numbness, paralysis, and even death (3). Therefore, the identification and protection of nerve tissue are crucial issues in many surgeries. Currently, the identification and functional monitoring of PNs in clinical practice primarily depend on electromyography, magnetic resonance imaging (MRI), computed tomography (CT), and ultrasound (US), as direct imaging methods for PNs are limited (4, 5). However, all these methods are challenging to achieve real-time localization and identification of the PN system during surgery.

In 2009, Professor Tsien firstly proposed the concept of OMI-guided surgery at the World Molecular Imaging Congress (6). This represented a milestone achievement in achieving real-time intraoperative identification and localization of target areas using fluorescent dyes. With over a decade of development, the clinical applications of several fluorescent agents, such as fluorescein sodium, methylene blue, and ICG, have become increasingly mature. This technology has successfully been employed in tumor labeling (7), vascular imaging (8, 9), ureteral imaging (10), sentinel lymph node identification (11–13), and cholangiography (14). However, limited clinical reports exist on PNs identification using this technique. This is primarily due to the absence of a nerve-specific fluorescent agent in current clinical practice.

According to reports, Rhodamine B shows strong specificity in achieving PNs fluorescence imaging (15). However, there is currently a lack of detailed research results regarding its usage, dose, and toxicity aspects. Therefore, our team conducted further investigations. Initially, we firstly performed nerve-specific validation of Rhodamine B on fresh human nerve tissue. Additionally, we examined its metabolism *in vivo* animals.

## 2 Materials and methods

### 2.1 Reagents

Rhodamine B [extinction coefficient is 85,000 L/(mol cm), quantum yield is 0.71], PBS, and the Weil’s Myelin Stain Kit were purchased from Acmec Biochemical Company in China, while the 4% paraformaldehyde solution was obtained from Boster Company in China. FBS, NeuroTrace blue, and DMEM cell culture medium were purchased from Thermo Fisher Scientific Company in the United States.

### 2.2 Animals and cells

All animals used in this study were approved by the Ethics Committee for Animal Welfare of Shanxi Medical University. Male C57 mice (22 g–24 g) required for the experiments were purchased from the Animal Experimental Center of Shanxi Medical University. Prior to surgery, animals were anaesthetized with 100 mg/kg ketamine.

The ND7/23 neuronal cells required for the experiment were obtained from the National Collection of Authenticated Cell Cultures. The culture medium consisted of 90 mL of DMEM cell culture medium supplemented with 10 mL of FBS. The cells were cultured under the following conditions: atmospheric composition: 95% air, 5% carbon dioxide; temperature: 37°C.

### 2.3 Fluorescence imaging system

**Figure 1:**
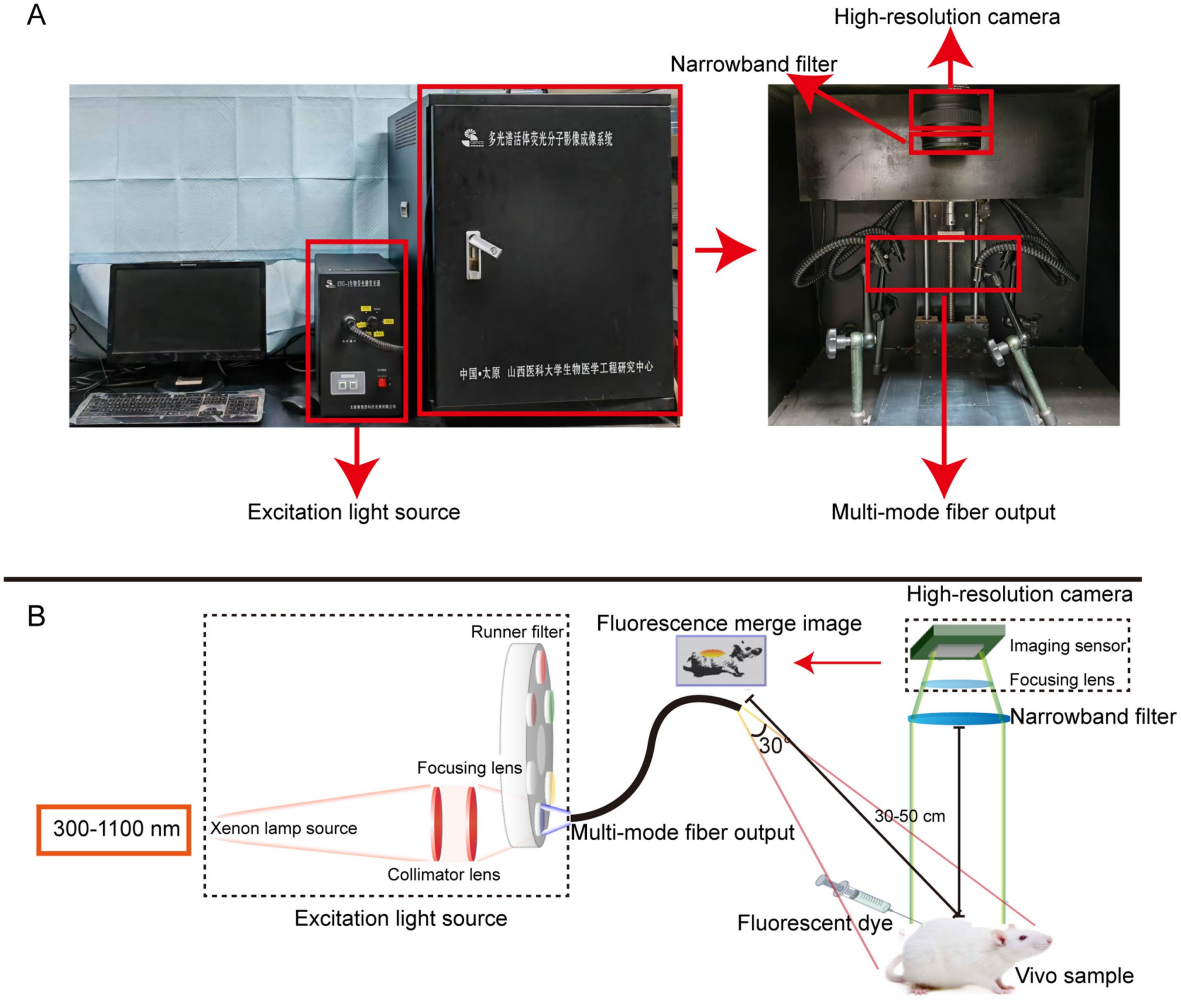
The self-developed Multi-modal Small Animal Live Imaging System. **(A)** Appearance of the imaging system. **(B)** Light path diagrams of the imaging system.

The *In Vivo* Xtreme Imaging System (Bruker BioSpin Corp., Billerica, MA) and the self-developed Multi-modal Small Animal Live Imaging System (Figure 1) were used to acquire fluorescent images of mice. The light source of the Multi-modal Small Animal Live Imaging System is a high-power xenon lamp with a spectral distribution ranging from 300 nm to 1,100 nm. It is coupled and output through a one-to-four multimode fiber, with the output ends distributed in four directions above the center of the illumination area, enabling uniform distribution of the light field during imaging. By selecting different wavelength excitation beams at the input end of the light source and using narrowband filters of different wavelengths at the receiving end of the camera, we can image the emitted fluorescence from different fluorophores. Additionally, the high-performance camera of this device collects fluorescence information, performs photoelectric conversion, transfers the data to a computer, and then uses Image-Pro Plus 7.0 software (Media Cybernetics, United States) for digital processing and analysis of the images.

### 2.4 Determining the optical properties of Rhodamine B in different formulations

1 nmol of Rhodamine B was added to 100 μL of PBS, NS, 5% GS, and FBS, respectively, to prepare Rhodamine B fluorescent solutions. Each fluorescent solution was then added to a 1 cm quartz cuvette, and the fluorescence spectra of the fluorophores were measured at room temperature using the Fiber Optic Spectrometer (AVANTES, Beijing). The results were normalized for subsequent analysis.

### 2.5 Study on the optimal dose and formulation of Rhodamine B

Rhodamine B was dissolved separately in 100 μL of PBS, NS, 5% GS, and FBS to prepare fluorescent dye solutions. Five different dose gradients were set: 1 nmol, 2 nmol, 4 nmol, 8 nmol, and 16 nmol, with each group consisting of 3 mice (*n* = 12). After anaesthetizing the mice, we followed the rinsing protocol established by the Barth and Gibbs (16) to expose the brachial plexus and sciatic nerve. The nerve tissue and background tissue were soaked in 100 μL of the fluorescent dye solution. After local incubation for 5 min, the treated area was gently dried using clean gauze. The area was then washed with 100 μL of blank formulation, repeating the process 9 times with each wash lasting 1 min. The area was dried again using clean gauze. Fluorescent images were acquired using the *In Vivo* Xtreme Imaging System (Bruker BioSpin Corp., Billerica, MA), and the fluorescence intensity data associated with the target regions in the captured images were collected for quantitative analysis. Based on the results, the optimal dose and formulation were selected. Finally, the mice were euthanized afterward.

### 2.6 Histological confirmation of mice nerve tissue

We dissolved the optimal dose of Rhodamine B in 100 μL of the recommended formulation. After repeating the staining steps mentioned above, the mice were euthanized, and the brachial plexus nerve and sciatic nerve, along with the surrounding tissues, were dissected. Frozen and paraffin sections with a thickness of 5 μm were prepared from above. For the paraffin-embedded sections, the first slide of each tissue was subjected to Rhodamine B fluorescence imaging (P1), the second slide was stained with H&E (P2), and the third slide was stained using the Weil’s Myelin Stain Kit according to the manufacturer’s instructions (P3). For the frozen sections, the first slide of each tissue was subjected to Rhodamine B fluorescence imaging (F1), the second slide was stained with H&E (F2), and the third slide was stained using NeuroTrace blue diluted in PBS (1:100) as per the manufacturer’s instructions (F3). Fluorescent images were captured for slides P1, F1, and F3 using a confocal fluorescence microscope (Leica, Germany) with Leica Application Suite X software (Leica, Germany), and pseudo-color was added. Images for sections P2, P3, and F2 were captured using an optical microscope (Leica, Germany) and 3DHISTECH’s Slide Converter (3DHISTECH, Hungary), and the images were processed using CaseViewer software (3DHISTECH, Hungary).

### 2.7 The localization experiment of neuronal cells

After resuscitating and passaging ND7/23 neuronal cells, we seeded them at a density of 2 × 10^5^ cells/mL, 500 μL, in confocal dishes and cultured them in the incubation environment until the cells adhered completely. The optimal dose of Rhodamine B was dissolved in 100 μL of the optimal formulation and co-incubated with the cells for 6 h and 24 h. The cells were then washed three times with PBS for 5 min each time. DAPI staining solution was added dropwise to the confocal dishes containing the cells and incubated for 5 min, followed by three washes with PBS for 5 min each. Subsequently, 300 μL of 4% paraformaldehyde solution was added for fixation, and the cells were scanned and imaged using confocal fluorescence microscopy.

### 2.8 Confirmation of fresh human nerve tissue

We dissolved the optimal dose of Rhodamine B in 100 μL of the optimal formulation. Nerve tissue from amputated limbs of patients who underwent limb removal surgery was collected along with the surrounding tissue and incubated in a fluorescent dye solution. Following incubation, the afore mentioned steps were repeated for washing. We used the self-developed Multi-modal Small Animal Live Imaging System to irradiate the *ex-vivo* tissue. Subsequently, we repeated the histological confirmation procedures for histological validation of fresh human nerve tissue.

### 2.9 Study on the metabolism of Rhodamine B in mice

We dissolved the optimal dose of Rhodamine B in 100 μL of the optimal formulation and administered it to mice through intravenous injection via the tail vein. The limbs of the mice were depilated, and the *In Vivo* Xtreme Imaging System was utilized to acquire fluorescence images. Data related to the fluorescence intensity in the target area of the images were collected for quantitative analysis. Mice were euthanized at different time points within a 3 months (m) period, and their hearts, livers, spleens, lungs, kidneys, brains, and nerve tissues were collected. Using the self-developed Multi-modal Small Animal Live Imaging System, fluorescent images of each organ at different time intervals were captured. Image-Pro Plus 7.0 software was used to add pseudo-color and obtain data related to relative fluorescence intensity in the target regions of the collected images. Subsequently, the tissues were fixed in formalin solution for 24 h, embedded in paraffin at a thickness of 5 μm, and subjected to H&E staining to observe the morphological changes in each tissue at different time points. Comparisons were made with the control group receiving the blank formulation and normal mice.

### 2.10 Statistical analysis

All count data were presented as mean ± standard deviation. GraphPad Prism 9.5 (GraphPad, United States) was used for statistical analysis. To assess the significance of differences in fluorescence intensity and SBR, a multifactor analysis of variance (ANOVA) was employed. In all analyses, *p* < 0.05 was considered statistically significant.

## 3 Result

**Figure 2:**
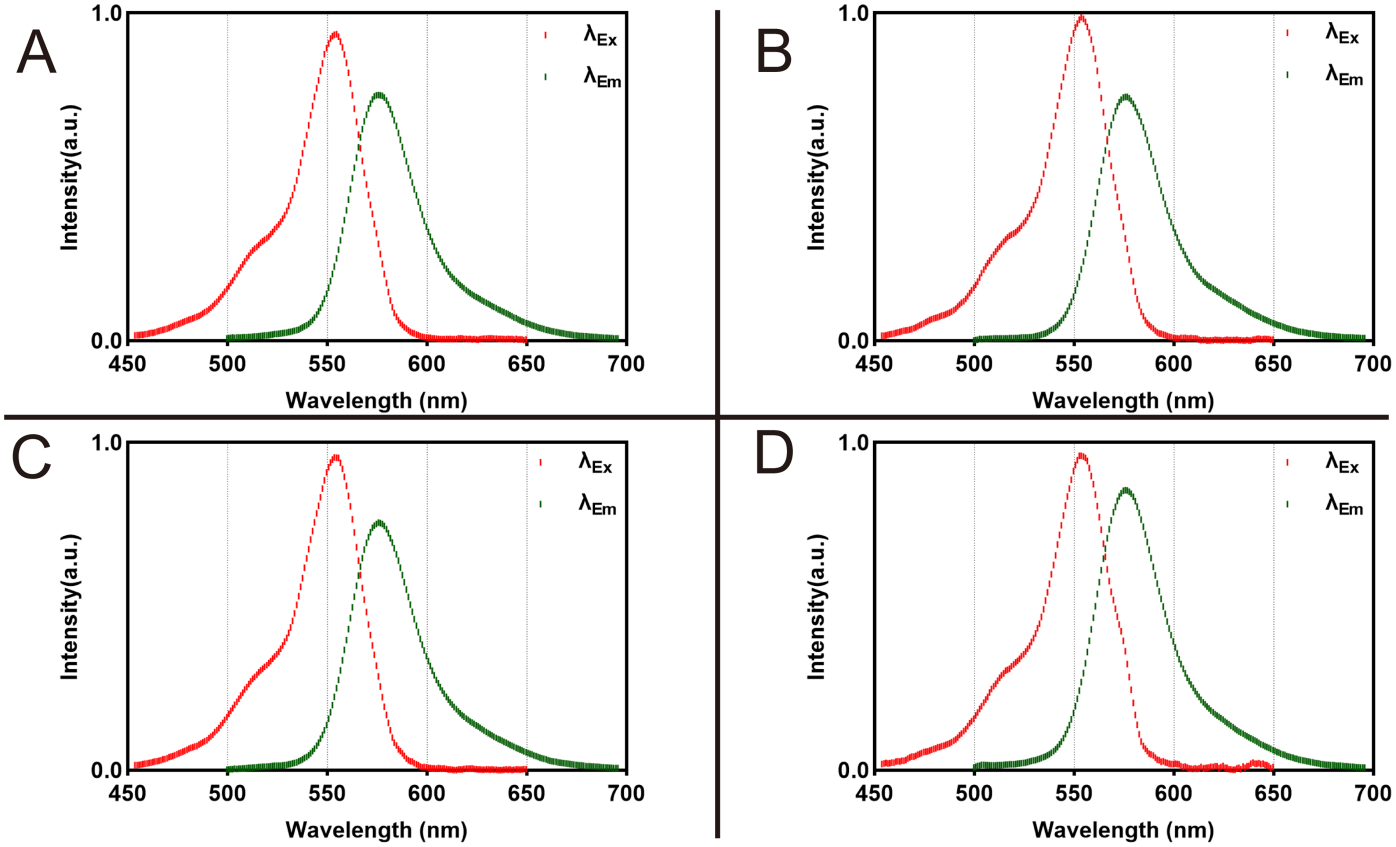
Fluorescence spectra of 1 nmol Rhodamine B dissolved in 100 μL different formulations. **(A)** Dissolved in PBS. **(B)** Dissolved in NS. **(C)** Dissolved in 5% GS. **(D)** Dissolved in FBS.

Rhodamine B exhibited an excitation peak at 554 nm and an emission peak at 576 nm in all four formulations (Figure 2 and Supplementary Tables S1–S4).

**Figure 3:**
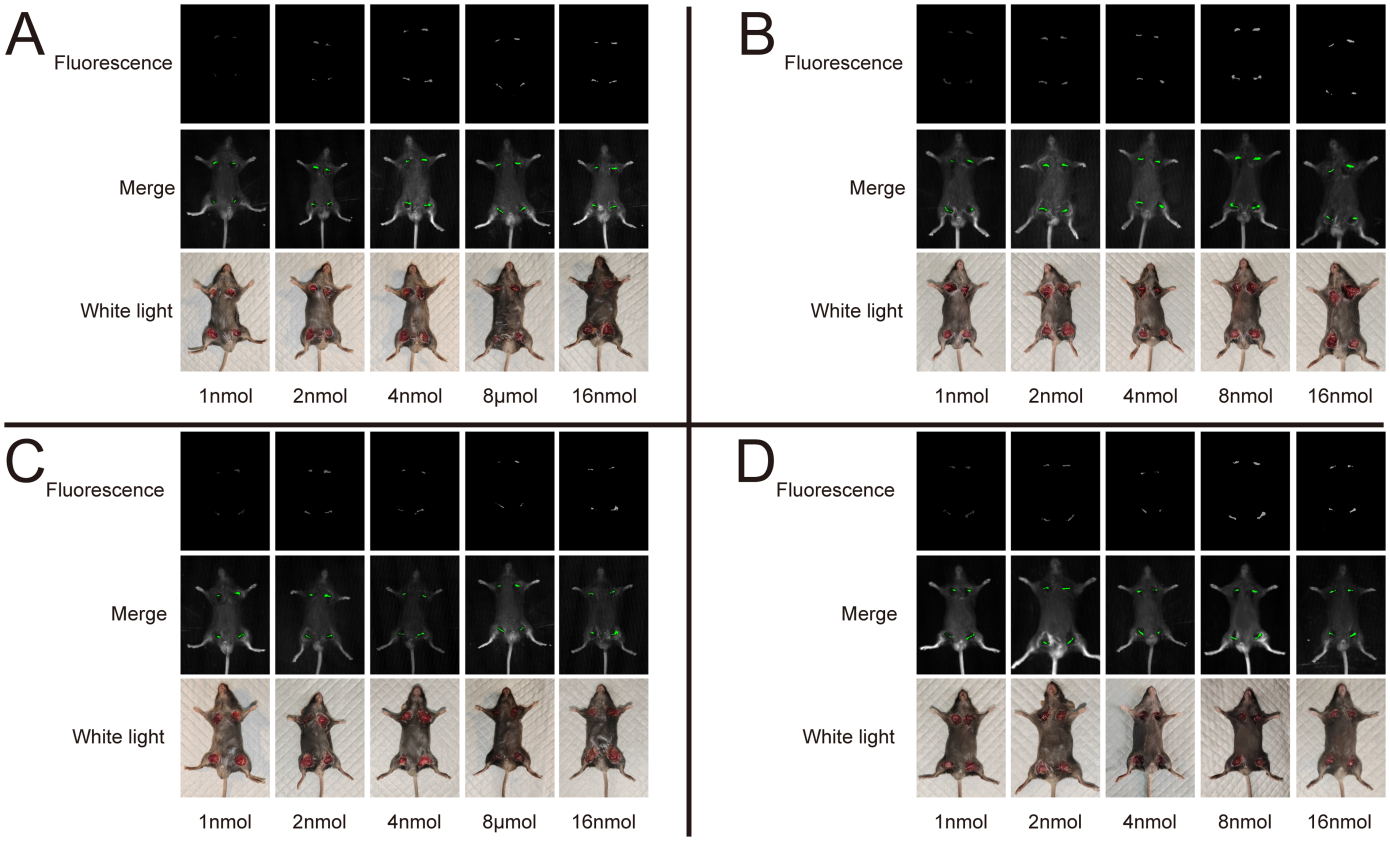
Representative image of different dose and formulation with Rhodamine B. We collected fluorescence images of nerve tissue, merge images, and white light images of mice in each group. All images acquired using the *In Vivo* Xtreme Imaging System (Bruker BioSpin Corp., Billerica, MA). **(A)** Dissolved in PBS. **(B)** Dissolved in NS. **(C)** Dissolved in 5% GS. **(D)** Dissolved in FBS.

**Figure 4:**
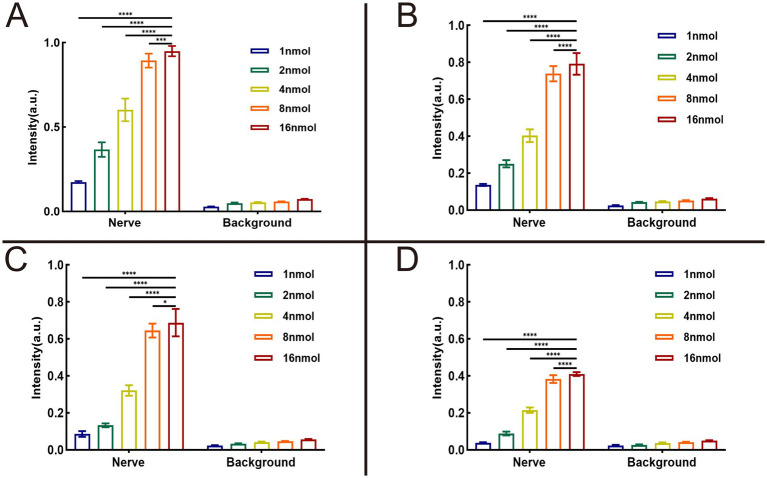
Results of optimal formulation and dose of Rhodamine B. The analysis results were collected from 3 mice (12 nerves) in each group. SBR = intensity of nerve tissue to intensity of background tissue ratio. The fluorescence intensity of nerve tissue and background tissue in different groups can be seen in **(A)** dissolved in PBS, **(B)** dissolved in NS, **(C)** dissolved in 5% GS, and **(D)** dissolved in FBS. * = *p* < 0.05, *** = *p* < 0.001, and **** = *p* < 0.0001.

**Figure 5:**
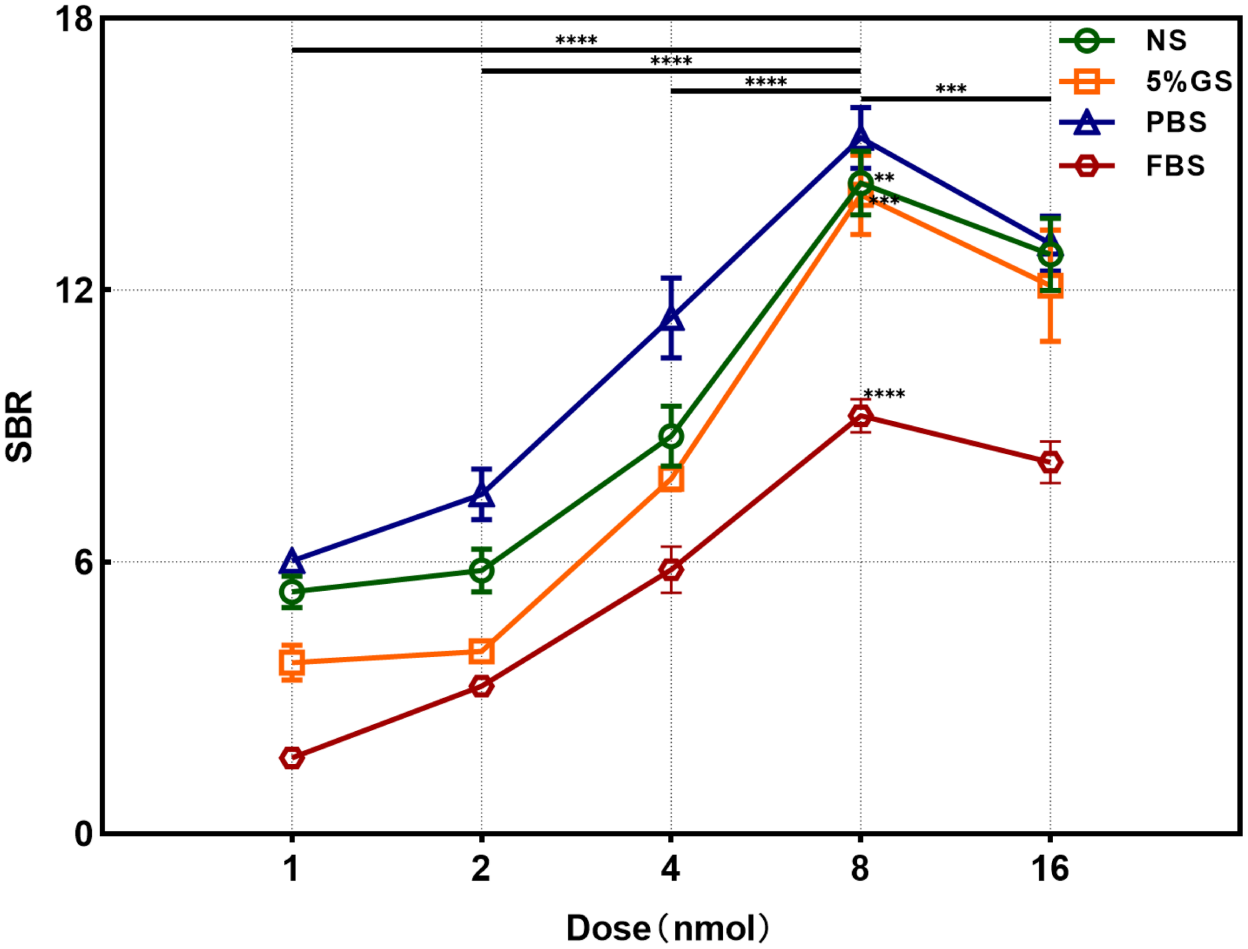
Analysis results of SBR in different formulation and dose groups. ** = *p* < 0.01, *** = *p* < 0.001, and **** = *p* < 0.0001.

We utilized the *In Vivo* Xtreme Imaging System for acquiring fluorescent images of brachial plexus and sciatic nerves in mice, which were incubated with varying doses of Rhodamine B and different formulations (Figure 3 and Supplementary Table S5). The fluorescence intensities of the nerve and background tissues were analyzed using the system’s built-in image processing tool, and then the SBR was calculated. The fluorescence intensity of the nerve tissue exhibited a positive correlation with the administered dose of Rhodamine B across the four formulations, displaying statistically significant differences (Figures 4A–D). Simultaneously, the fluorescence intensity of the background tissue exhibited an increase as well (Figures 4A–D). Through the calculation of the SBR (Table 1 and Figure 5), it was determined that within the 1–8 nmol range, the SBR displayed a direct proportionality to the administered dose of Rhodamine B. When the administered dose reached 8 nmol, the PBS group exhibited the highest SBR value of 15.37 ± 0.68. Notably, this result demonstrated a statistically significant difference when compared to the NS group (14.37 ± 0.70), 5% GS group (14.11 ± 0.88), and FBS group (9.23 ± 0.36). Furthermore, statistical significance (*p* < 0.0001) was also observed when comparing these results with other Rhodamine B doses in the same formulation: 1 nmol (6.02 ± 0.10), 2 nmol (7.50 ± 0.56), 4 nmol (11.39 ± 0.88), and 16 nmol (14.14 ± 0.51). These findings suggest that PBS is the optimal formulation choice for achieving real-time OMI in the PNs using Rhodamine B, with an optimal dose of 8 nmol.

**Table 1:** The SBR in different formulations.

Dose (nmol)					
Formulations	1	2	4	8	16
PBS	6.02 ± 0.10	7.50 ± 0.56	11.39 ± 0.88	15.37 ± 0.68	13.03 ± 0.61
NS	5.34 ± 0.35	5.82 ± 0.47	8.78 ± 0.67	14.37 ± 0.70	12.79 ± 0.80
5% GS	3.78 ± 0.38	4.03 ± 0.19	7.84 ± 0.25	14.11 ± 0.88	12.10 ± 1.23
FBS	1.67 ± 0.23	3.26 ± 0.17	5.83 ± 0.51	9.23 ± 0.36	8.20 ± 0.46

**Figure 6:**
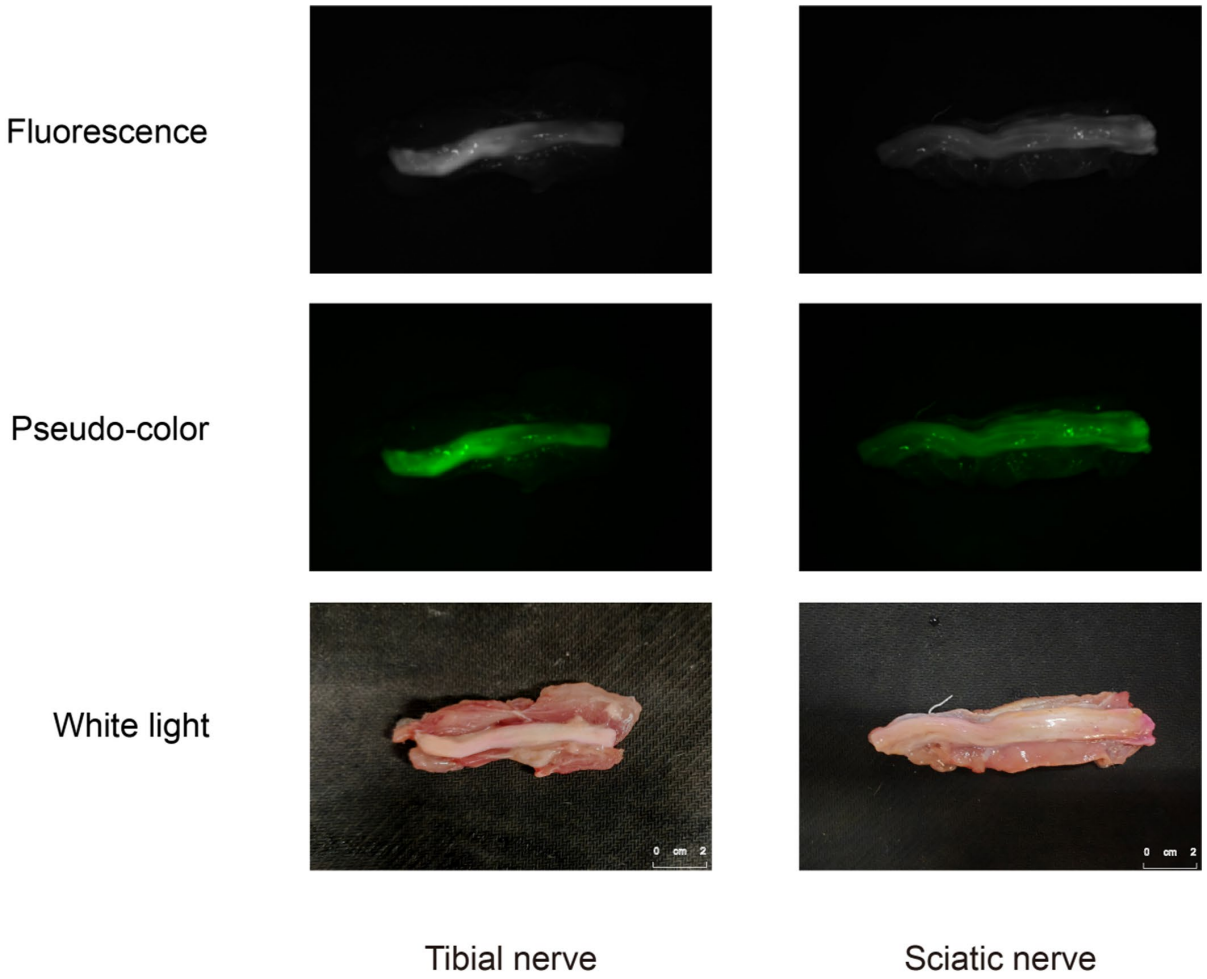
Fluorescence images, pseudo-color images, and white light images of human *ex-vivo* nerve tissue.

**Figure 7:**
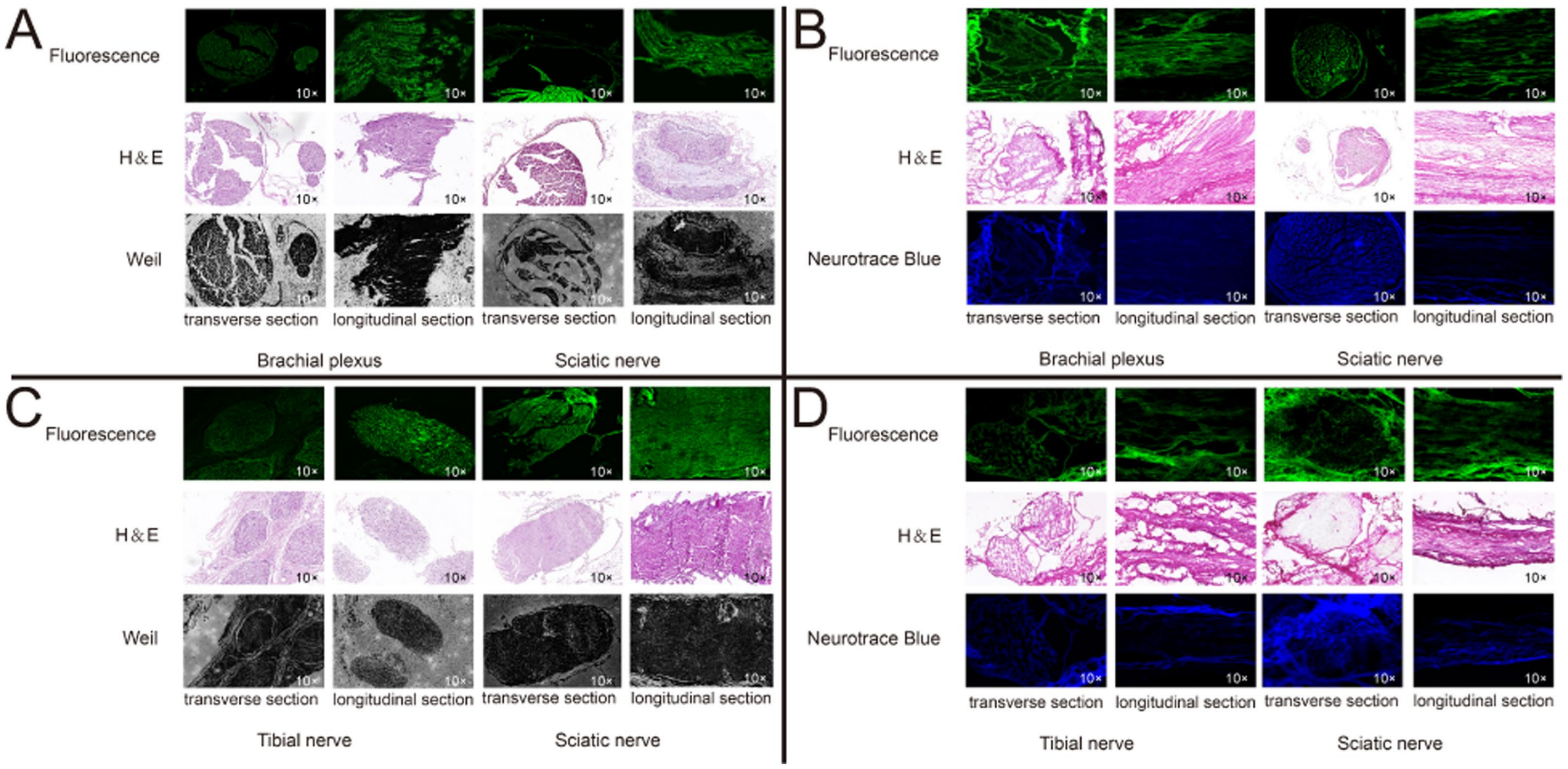
Histological confirmation of nerve tissue. We confirmed the brachial plexus and sciatic nerve in mice, and the isolated tibial nerve and sciatic nerve in human from transverse and longitudinal section, respectively, using paraffin and frozen sections, respectively. Each set of sections were derived from three consecutive sections. **(A)** Mice nerve tissue, paraffin section. **(B)** Mice nerve tissue, frozen section. **(C)** Mice nerve tissue, paraffin section. **(D)** Human nerve tissue, frozen section.

Through the collection of *ex-vivo* tissue fluorescent images, distinct and robust fluorescent signals were observed specifically in the regions corresponding to the nerve tissue, whereas the background tissue exhibited minimal fluorescence signal (Figure 6). The nerve tissue is rich in Nissl substance and myelin sheaths. To label the Nissl substance, we utilized the NeuroTrace blue fluorescent stain and applied a blue pseudo-color to the corresponding fluorescent images of the P3 sections. Moreover, to visualize the myelin sheaths, we employed the Weil’s Myelin Stain Kit, which produces a black appearance upon binding. For the fluorescent images of sections F1 and P1, a green pseudo-color was applied. The discrepancies between the nerve tissue and the surrounding tissue were validated by performing H&E staining. Upon examination of the fluorescent images of Rhodamine B, a strong correspondence was observed between the fluorescent regions and the nerve tissue localization, which was further corroborated by H&E staining, Weil’s Myelin Stain Kit, and NeuroTrace blue staining (Figures 7A,B). This reaffirms the specific affinity of Rhodamine B towards mouse nerve tissue. This specificity is also applicable to fresh human nerve tissue (Figures 7C,D), suggesting the clinical potential of Rhodamine B for PNs fluorescence imaging.

**Figure 8:**
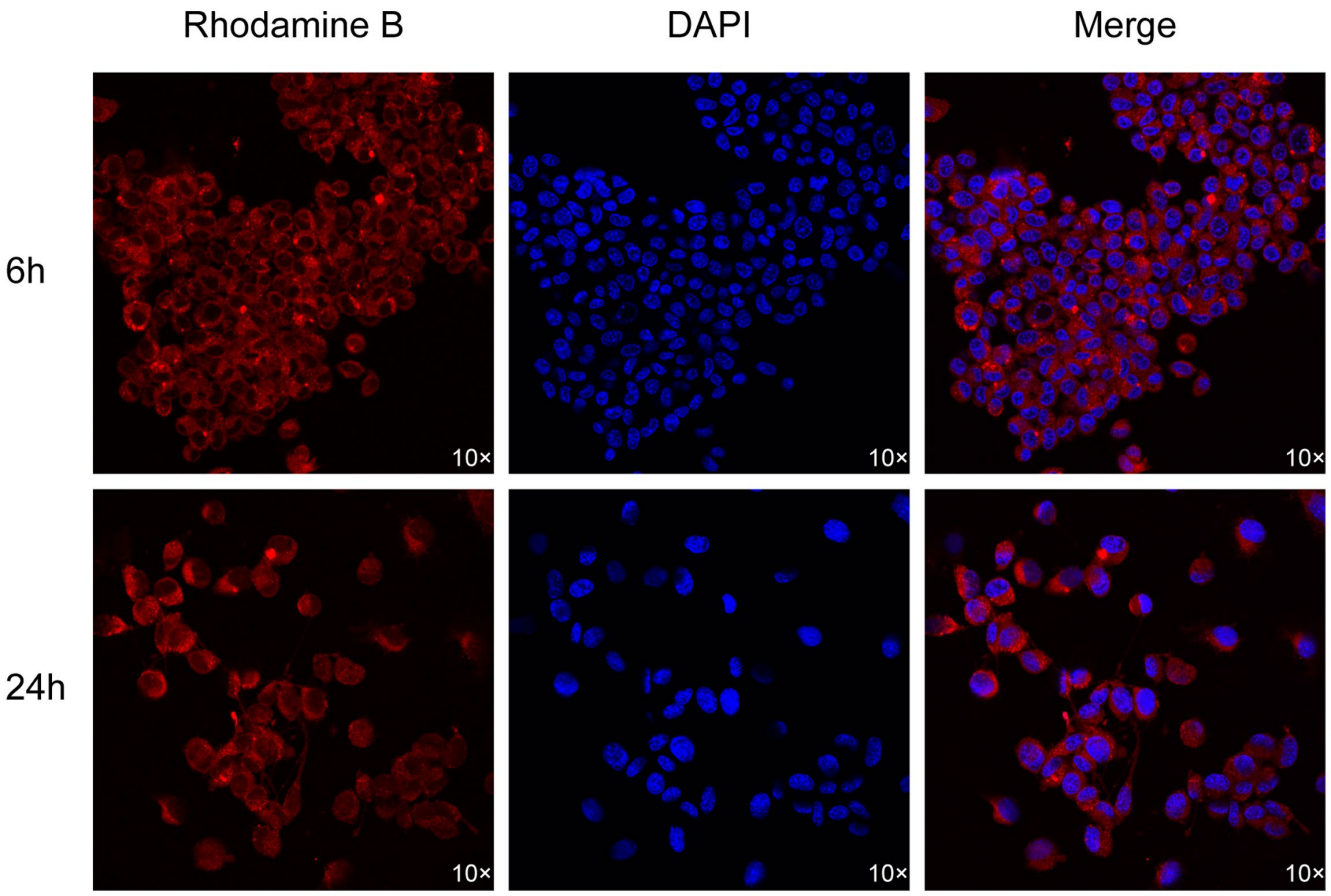
Fluorescence microscopy image of ND7/23 neuronal cells.

As shown in Figure 8, the staining effect of Rhodamine B on ND7/23 neuronal cells is illustrated. We observed that the fluorescence of Rhodamine B is primarily concentrated on the cell membrane of neuronal cells, with no obvious red fluorescence observed within the cells. This indicates that the staining site of Rhodamine B is localized on the cell membrane of neuronal cells.

**Figure 9:**
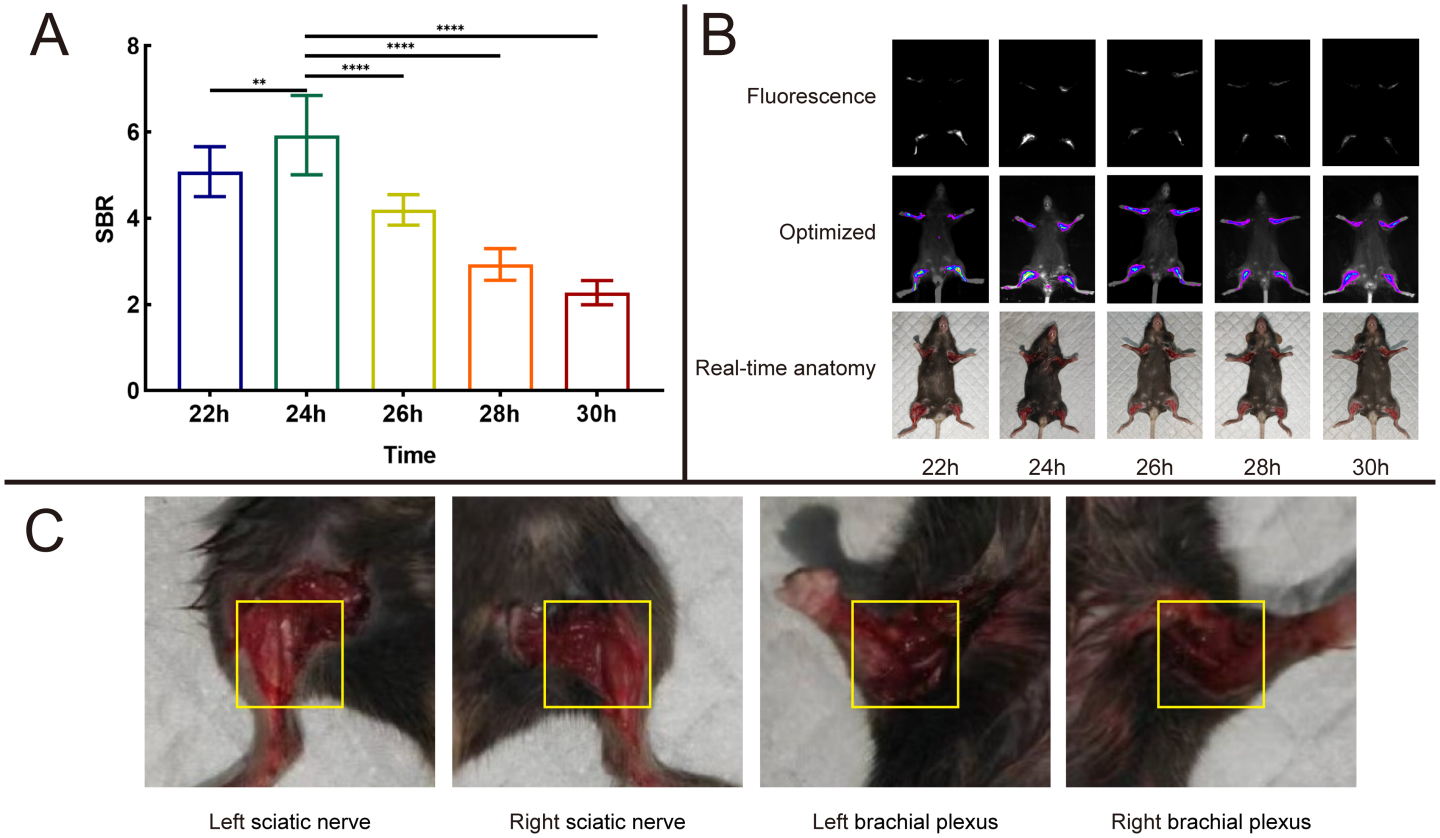
Result of time dependent imaging. **(A)** Analysis results of SBR in different time of 8 nmol Rhodamine B dissolved into 100 μL PBS and injected through a mice tail vein. **(B)** Fluorescence images, optimized images, and real-time anatomy images. **(C)** Anatomical images of mouse nerve tissues result at 24 h post-operation based on fluorescence signal guidance.

An intravenous injection of 8 nmol of Rhodamine B, which was dissolved in 100 μL of PBS, was administered to the mice via the tail vein. After depilation of the mouse limbs, fluorescent images were acquired using the *In Vivo* Xtreme Imaging System, and data related to the fluorescence intensity in the target area were collected for quantitative analysis. By calculating the SBR (Table 2), we found that at 24 h, the SBR of the nerve tissue reached it speak (5.93 ± 0.92), which was significantly higher compared to other time points (22 h: 5.08 ± 0.58, 26 h: 4.20 ± 0.35, 28 h: 2.93 ± 0.37, 30 h: 2.28 ± 0.28) (Figure 9A). Guided by the fluorescent images, we anesthetized the mice and dissected the target area (Figures 9B,C). We found that the brachial plexus and sciatic nerve of the mice corresponded well with the area showing the fluorescence signal in the fluorescent images.

**Table 2:** The SBR in formulate of PBS in different time.

Time (h)	22	24	26	28	30
SBR	5.08 ± 0.58	5.93 ± 0.92	4.20 ± 0.35	2.93 ± 0.37	2.28 ± 0.28

**Figure 10:**
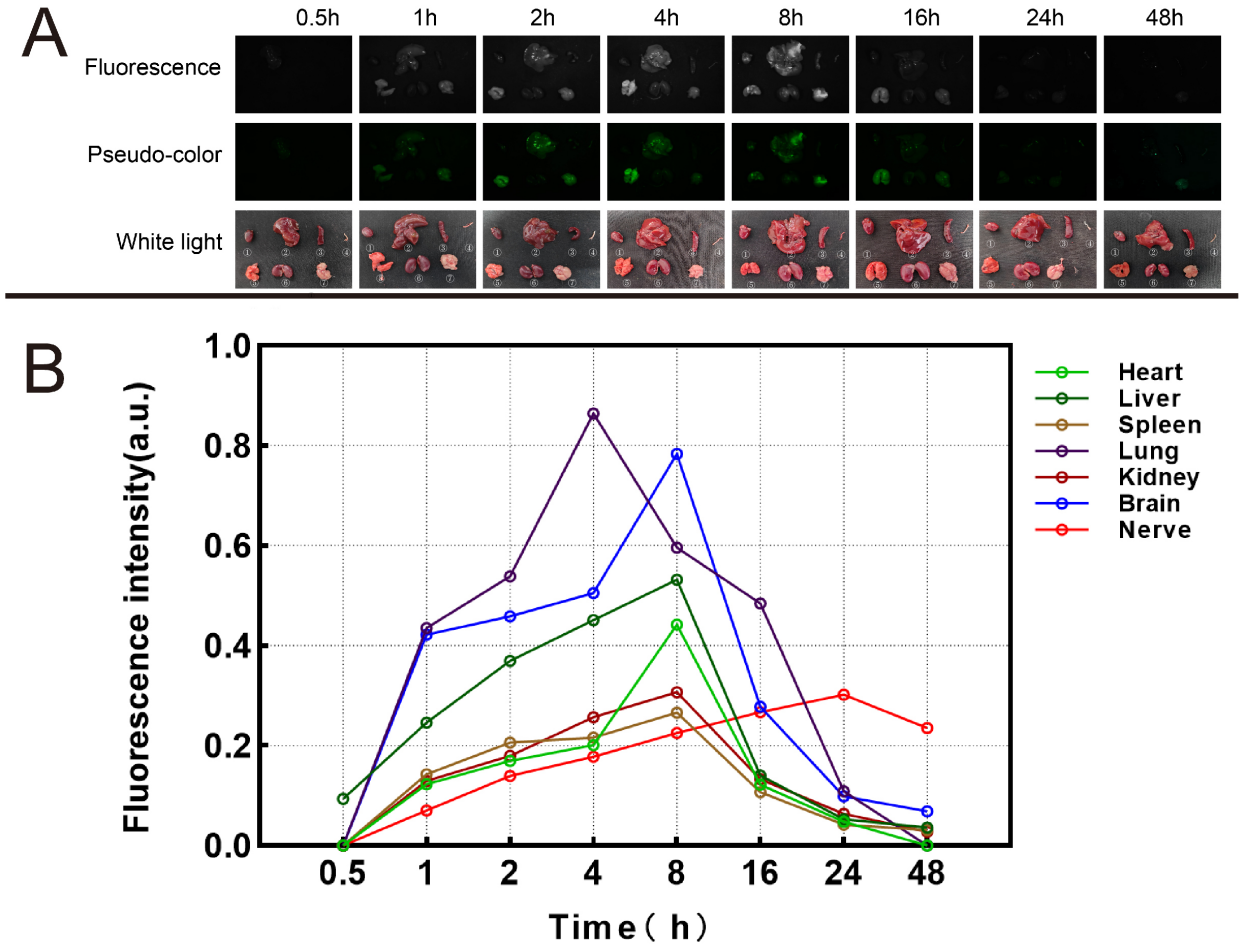
Metabolic results of Rhodamine B in mice. **(A)** Fluorescence images, pseudo-color images, and white light images of different organs at different time. ① Heart. ② Liver. ③ Spleen. ④ Nerve. ⑤. Lung. ⑥ Kidney. ⑦ Brain. **(B)** Fluorescence intensity changes of different organs at different time.

Mice were euthanized at different time points, including 0.5 h, 1 h, 2 h, 4 h, 8 h, 16 h, 24 h, 48 h, 1 w, 2 w, 1 m, 2 m and 3 m. We used the self-developed Multi-modal Small Animal Live Imaging System to observe the fluorescent signal images of various organs (Figure 10 and Supplementary Table S6). If no fluorescent signal was detected in certain organs, the fluorescence intensity value was represented as 0. Notably, the liver displayed a fluorescence signal as early as 0.5 h, which subsequently reached it speak intensity at 4 h. In contrast, the remaining tissues commenced exhibiting fluorescence signals from 1 h onwards. Moreover, the fluorescence signals within the heart, spleen, lungs, kidneys, and brain attained their maximum intensity at 8 h. Notably, the fluorescence signal within the nerve tissue exhibited its peak intensity at 24 h and sustained for a minimum of 48 h. This duration permits sufficient time for the identification, isolation, and protection of nerve tissue during surgical interventions.

**Figure 11:**
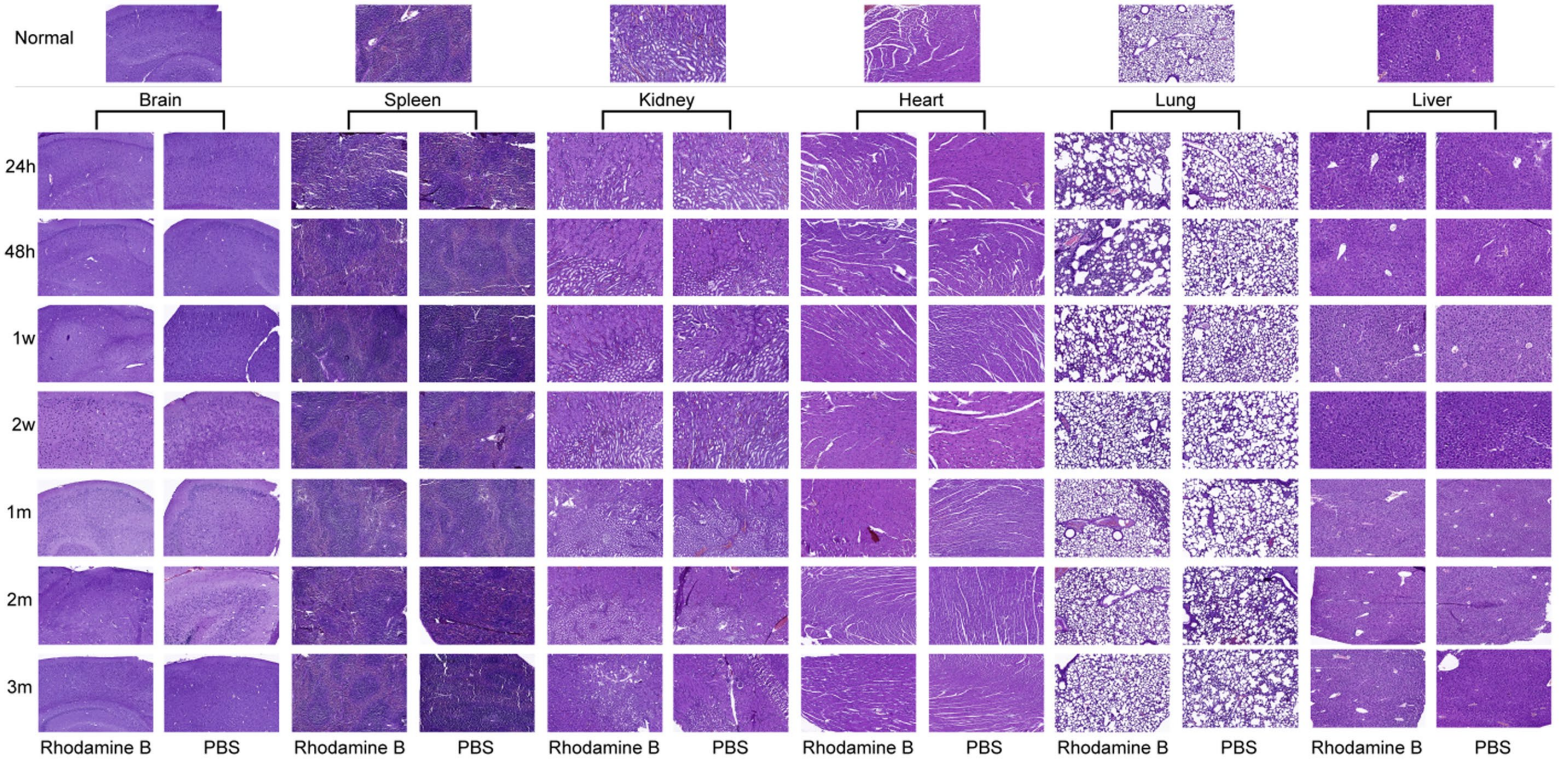
Histological changes of different organs at different time after 100 μL Rhodamine B solution and PBS were injected into mice via the tail vein H&E.

To account for the potential toxicity of Rhodamine Bon diverse organs, a control group administered with PBS was established for subsequent investigations. By comparing the morphologies of various tissues stained with H&E after injecting the same amount of Rhodamine B solution or PBS in two groups of mice, they were also compared with corresponding tissue sections from normal mice (Figure 11). Notably, both experimental groups demonstrated vascular dilation and alveoli fusion in the lungs at 24 h post-injection. However, these pathological indications gradually ameliorated and reverted to their baseline levels within 2 w. Additionally, no signs of acute or chronic inflammatory responses were observed in the other examined organs.

## 4 Discussion

Iatrogenic nerve injury is a major surgical complication that significantly affects the patient’s quality of life. Currently, commonly used imaging techniques like US, CT, and MRI have limited sensitivity and accuracy in detecting subtle lesions and are not suitable for providing real-time feedback or guidance during surgery. However, OMI technology offers a potential solution by utilizing fluorescent substances or exogenous fluorescent compounds with specific optical imaging equipment. This technology is becoming more common in surgical procedures. However, the bundled structure of nerve fibers presents challenges in achieving fluorescent imaging due to difficulties in staining the fluorescent dyes. Over the years, researchers have been dedicated to developing and investigating neuro-fluorescent dyes.

The extinction coefficient is a parameter that describes the molecular ability to absorb light, determining the light absorption efficiency of a fluorophore at a specific wavelength. A higher extinction coefficient means that a strong fluorescence signal can be produced under lower excitation light intensity, which is crucial for reducing phototoxicity and improving the SBR in imaging. The quantum yield describes the efficiency with which a fluorescent molecule emits fluorescence after absorbing light energy. A fluorophore with a high quantum yield can produce brighter fluorescence signals in neural tissue, which helps to enhance the contrast and sensitivity of imaging (17, 18). Rhodamine B has a high extinction coefficient [85,000 L/(mol cm)], making it very effective in bioimaging, especially in applications requiring high sensitivity and low light conditions. Additionally, Rhodamine B has a high quantum yield (0.71), enabling it to produce intense fluorescence signals under a fluorescence microscope, facilitating the clear observation of subtle neural structures. We have calculated the Stokes shift of Rhodamine B, which refers to the difference in peak positions between the absorption spectrum and the fluorescence emission spectrum. The Stokes shift of Rhodamine B (*λ*_Em_ − *λ*_Ex_) is 22 nm, which helps to reduce the overlap between the excitation and emission light, thereby enhancing the clarity of imaging and reducing background interference.

Rhodamine B, as a high-efficiency fluorescent dye, has also shown considerable effects in tissue identification and disease diagnosis. It was reported that Quan et al. (19) applied Rhodamine B on the knee cartilage to explore the molecular transport rules in different layers of cartilage by constructing different operating paths to guide the treatment and defect repair of osteoarthritis. Molecular fluorescence probes YIGSR-RhB and RGD-RhB constructed by Liu et al. (20) based on Rhodamine B also showed high fluorescence signal accumulation in mouse 4T1 breast cancer and B16F10 melanoma, which can greatly improve the accuracy of tumor targeting recognition. The research of Gibbs et al. (15) also provided the basis for our research.

In this study, we selected four commonly used formulations in clinical and animal experiments: PBS, NS, 5% GS, and FBS. We investigated the feasibility and effectiveness of Rhodamine B for intraoperative nerve fluorescence imaging using animal experiments and fresh human tissue experiments. Previous research by the Gibbs et al. (15) confirmed the specific binding of Rhodamine B to nerves. However, no relevant study has been conducted on its photophysical properties, doses, systemic metabolism, or toxicity. Building on their research, we experimentally validated the optical characteristics of Rhodamine B. Subsequently, we explored the optimal dose and formulation for Rhodamine B based on the validation results. Additionally, we further investigated the specific binding of Rhodamine B to nerve tissue using fresh human *ex-vivo* nerve samples. Finally, we explored the *in vivo* metabolism of Rhodamine B in various tissues using animal experiments. Based on the pathological sections of different tissues at different time intervals, we conducted preliminary assessments of the toxicity of Rhodamine B. Our study offers additional information for the utilization of Rhodamine B in the field of realizing real-time OMI in PN tissue.

To ensure fairness and consistency in the experiment, we ensured that Rhodamine B in each solvent received the same dose treatment. Figure 1 shows the data we obtained. From it, we can observe that although the *λ*_Ex_ and *λ*_Em_ of Rhodamine B in these four solvents are the same, its fluorescence intensity shows significant differences. This is mainly related to the recognition and positioning of the quartz cuvette and the capture of optical signals by the Fiber Optic Spectrometer during the test. The core goal of this test is to analyze the peaks in the fluorescence spectrum of Rhodamine B to determine its *λ*_Em_ and *λ*_Ex_. Therefore, although the fluorescence intensity is different in each group, this does not change our measurement results of *λ*_Em_ and *λ*_Ex_. In the field of fluorescence spectrum analysis, scholars usually pay more attention to wavelength rather than intensity because wavelength is a direct manifestation of fluorescence characteristics. At the same time, during the analysis and review of experimental data, such differences in intensity are often ignored because they do not affect our interpretation of *λ*_Em_ and *λ*_Ex_ of fluorescent dyes.

As a zwitterionic fluorescent dye, Rhodamine B can specifically bind to nerve tissue. However, it should be emphasized that, due to its own physicochemical properties such as high permeability, long retention time, and lipophilicity, in the experimental context, Rhodamine B will inevitably bind to background tissue to some extent in addition to its affinity for nerve tissue (21–23). In fact, this phenomenon of binding to background tissue is not an isolated feature unique to Rhodamine B but a common characteristic among many fluorescent dyes (24).

In a series of carefully planned and rigorously executed experimental processes, we carried out meticulous observations and detailed recordings of the fluorescence signal performance after the interaction of Rhodamine B with nerve tissue and background tissue. When the nerve tissue was exposed to the Rhodamine B solution, we were delighted to observe that the nerve tissue could emit a highly intense and prominent fluorescence signal, and the intensity of this signal was sufficient to be clearly distinguished and differentiated in the experimental detection system. In comparison, the fluorescence signal emitted by the background tissue was much weaker, forming a sharp contrast between the two. This significant difference provides solid and powerful experimental support for us to accurately distinguish the nerve tissue from the surrounding background tissue in the complex biological tissue environment, thus creating favorable conditions for the precise localization and identification of nerve tissue.

We further found that there is a close and definite correlation between the intensity of the fluorescence signal and the administered dose of Rhodamine B. With the gradual increase of the Rhodamine B dose, the fluorescence signal intensity of the nerve tissue also shows a corresponding upward trend. After careful data collection and rigorous statistical analysis, we accurately determined that when the dose of Rhodamine B reaches approximately 8 nmol, the SBR of the nerve tissue fluorescence climbs to its peak state. The clear definition of this dose-signal relationship is of crucial guiding value for us to optimize the use dose of Rhodamine B in subsequent practical application scenarios to obtain the most ideal nerve tissue imaging effect.

Going deeper into the microscopic structure level of nerve tissue, the myelin sheath, as a key structural component widely existing in the central nervous system and the peripheral nervous system, has the core function of tightly surrounding the axons of nerve cells, building an insulating barrier, and effectively blocking the disordered transmission of nerve impulses between adjacent neuronal axons (25, 26). During our detailed observation of neuronal cells using advanced fluorescence microscopy technology, we clearly saw that the staining sites of Rhodamine B in neuronal cells show a highly concentrated distribution pattern, mainly concentrated in the cell membrane region. At the same time, almost no fluorescence signal of Rhodamine B was observed inside the cells, which strongly suggests that there may be a specific interaction mechanism between Rhodamine B and the cell membrane.

To further verify the binding specificity of Rhodamine B to nerve tissue at the tissue level, we carefully prepared nerve tissue slices and carried out meticulous observations. By comparing and analyzing with the myelin sheath localization information provided by the Weil’s Myelin Stain Kit, we were pleasantly surprised to find that the fluorescence area of Rhodamine B almost perfectly overlaps with the localization of the myelin sheath. This highly consistent result provides more direct and convincing evidence for our previous speculation and further strongly supports the hypothesis that Rhodamine B may have the ability to specifically bind to the myelin sheath in nerve tissue. When referring to the important research results of other relevant research teams, we noted that the Tsien et al. (27) clearly revealed in their research that the laminin abundant in the peripheral nerve myelin sheath can specifically bind to the nerve-specific peptide sequence NP41, thus achieving the fluorescence imaging of nerve tissue. Gu’s et al. (20) also found that Rhodamine B has the ability to bind to the laminin receptors on melanoma cells and breast cancer cells and shows good fluorescence imaging effects *in vitro* cell experiments and tumor-bearing mouse models. Considering these research findings comprehensively, we have sufficient grounds to speculate that laminin is very likely to be the potential key binding target in the specific interaction process between Rhodamine B and nerve tissue. This speculation opens up a new research path for us to further explore the mechanism of action of Rhodamine B in nerve tissue and also provides an important theoretical basis for designing more accurate and efficient nerve imaging experiments in the future. Although the current experimental results and related research all strongly point to the possibility that laminin is the binding target of Rhodamine B, we also clearly recognize that to conclusively confirm this hypothesis, more in-depth and systematic research work is still needed.

Through metabolic studies in mice, we found that the SBR of nerve tissue reached maximum at 24 h. Guided by fluorescent imaging, we successfully dissected the brachial plexus and sciatic nerve of the mice. This also confirms the feasibility of precise localization and identification of nerve tissue in surgical procedures using this research approach. By measuring the metabolism within different organs of mice at different time points, we found that at 24 h, the fluorescence intensity of mouse nerve tissue reached its maximum, consistent with the maximum SBR value of mouse nerve tissue measured during this time period. This also suggests that in this study, the optimal staining and injection time for Rhodamine B is 24 h.

Research has shown that high doses of Rhodamine B administered to experimental animals can cause skin tearing, liver atrophy, and tumors (28–32). Acute excessive exposure of human to nebulized Rhodamine B can result in temporary mucosal and skin irritation, which typically subsides within 24 h (33). Through H&E staining, we confirmed that mice exhibited pulmonary alveolar fusion within 24 hat a dose of 8 nmol, but this phenomenon gradually diminished overtime and completely disappeared by 2 w. No abnormalities were observed in the concurrent myocardial tissue. This phenomenon is mainly due to the fact that Rhodamine B may affect vascular permeability and thus affect the function of blood vessels (34). Intravenous injection causes a sudden increase in blood volume, resulting in elevated pulmonary venous pressure, dilation of blood vessels, and fluid flowing into the lungs at a rate that exceeds the lymphatic drainage capacity. Additionally, increased tissue water content affects gas exchange between the alveoli and tissues, ultimately resulting in pulmonary alveolar wall damage and fusion (35–37). As time progresses and the mouse’s blood circulation stabilizes, this symptom will gradually alleviate until it disappears. Following the injection of the fluorescent dye solution via the tail vein, the liver was the first organ to exhibit fluorescence. Fletcher’s study (38) on rats demonstrated that subcutaneous injection of high dose Rhodamine B commonly resulted in liver tissue atrophy, congestion, hemosiderin accumulation, increased Kupffer cell count, amyloid-like degeneration, nuclear degeneration, and vacuolar degeneration of liver cells. These findings contrast with our experimental results. Additionally, no histological changes were observed in the kidneys or spleen, which further contrasts with the results of the aforementioned study. The main reason is that the doses used by that team (50–200 mg/kg/week) are much higher than our intended usage dose. Byer’s et al. (39) showed a study that demonstrated that low doses (1 mg/kg) of Rhodamine B do not exhibit hepatotoxicity. Therefore, in practical applications, it is necessary to strictly control the dose of Rhodamine B within a safe range to avoid causing irreversible damage to various organs in the body. By observing the pathological sections of each organ of mice, we did not find that Rhodamine B had chronic pathological damage to each organ of mice.

At present, in the field of fluorescence imaging-guided surgery, the fluorescence range varies from the visible light to the near-infrared (NIR) region, depending on the applied fluorescent dyes (40). Compared with fluorescent dyes in the visible light range, NIR fluorescent dyes have their unique advantages, such as lower tissue autofluorescence, higher SBR, and deeper penetration into biological tissues (41–43). In the earliest studies, researchers used fluorescent dyes to stain individual axon bundles through anterograde or retrograde axonal transport (44–47). Subsequently, researchers attempted to achieve fluorescence imaging of certain nerve tissues in the thoracic and abdominal cavities using indocyanine green (ICG). However, ICG had limitations such as low quantum yield, poor optical stability, and lack of targeting capabilities, making it difficult to distinguish accurately from surrounding tissues in practical applications (48–50). Several studies on distyrylbenzene and its derivatives have found that increasing the number of double bonds affects the optical properties of fluorescent dyes, shifting their spectral range towards the NIR region (51, 52). Gibbs’s et al. (53) found that the fluorescence penetration depth of oxazine-4 in visible light was less than 1 mm. However, its modified derivative, LGW01-08, exhibited a fluorescence penetration depth of up to 2 mm in the NIR region. Unfortunately, due to limitations in experimental equipment, we were unable to experimentally verify the aforementioned research findings.

Based on the aforementioned studies, Choi’s et al. (54) proposed that ideal neuronal fluorescent dyes should possess the following characteristics: a molecular weight below 500 Da to ensure optimal penetration through the blood–brain barrier, and excitation and emission wavelengths within the NIR range. Therefore, in recent years, the development of nerve-specific fluorescent dyes has predominantly focused on two directions. One approach involves modifying the chemical structure of existing neuronal fluorescent dyes to shift their fluorescence spectra into the NIR range. The other approach involves combining existing NIR fluorophores with nerve-specific probes to create new compounds. In the latter studies, dyes such as FAM-NP401, Hs1a-FL, and BChE-IRDye have demonstrated excellent neuronal fluorescence effects in animal experiments (55–57). Compared with other agents such as FAM-HNP401, Hsp1a-FL, oxazine and phenoxazine, Rhodamine B has advantages in practical application (53, 57–59). Rhodamine B has a strong fluorescent emission in the visible range, which makes it very popular in traditional fluorescence imaging. Its excitation and emission wavelengths are suitable for most fluorescence imaging systems, which makes it easy to use in laboratory and clinical settings. Rhodamine B usually has good light stability, which means that it can maintain a fluorescent signal for a long time under continuous light, which is especially important for long surgery or imaging procedures. Rhodamine B is a lower cost fluorophore than other novel fluorophore, which makes it more attractive in resource-limited environments. Rhodamine B has been extensively studied for many years, with a wealth of literature and practical experience, which provides a solid scientific basis for the use of it.

**Figure 12:**
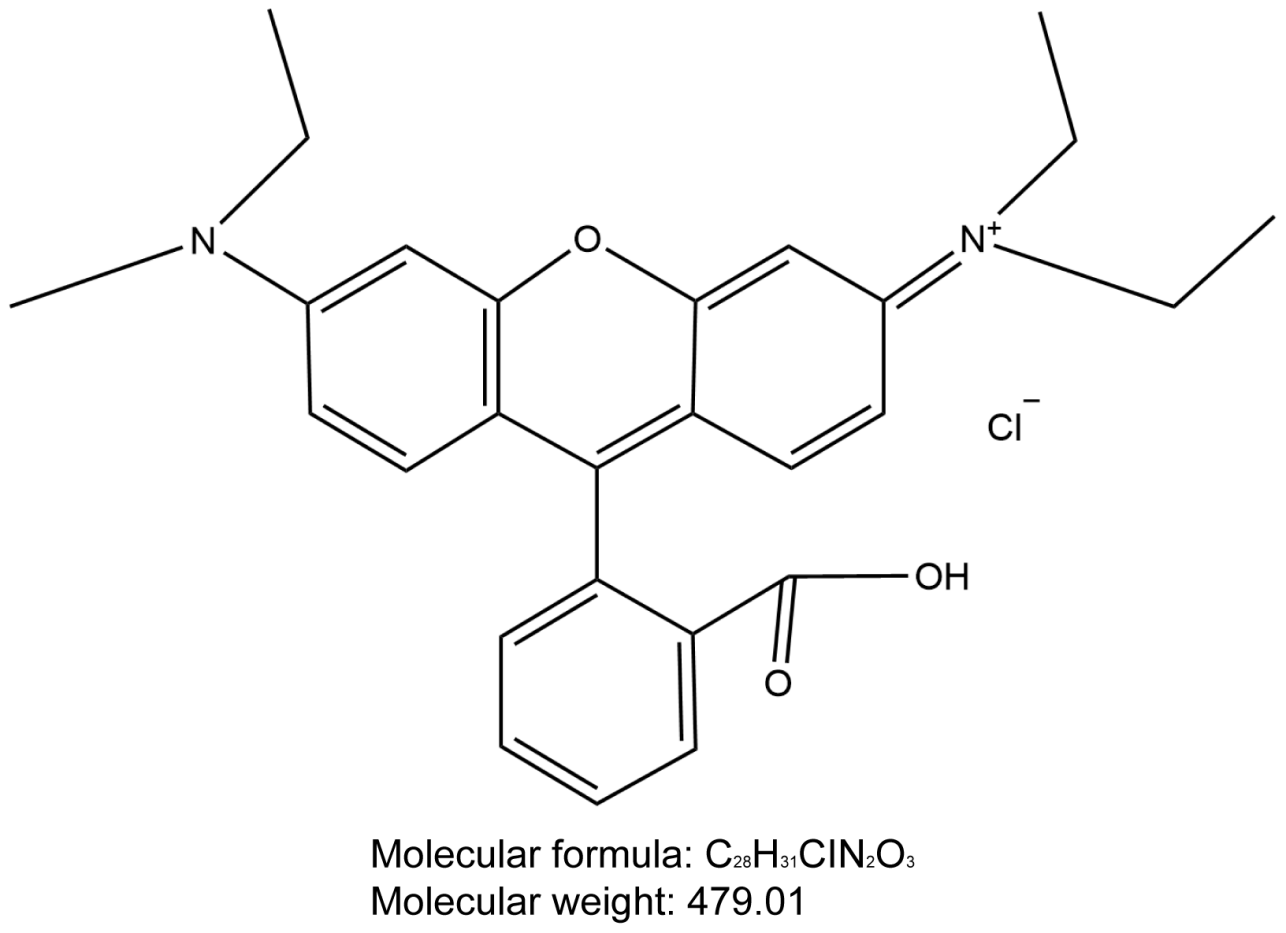
Chemical structure of Rhodamine B. The molecular formula for Rhodamine B is C_28_H_31_ClN_2_O_3_ and the molecular weight is 479.01.

Through our research, we have discovered that Rhodamine B (Figure 12) could potentially a new tool for intraoperative nerve identification for surgeons. Compared to traditional surgery, the innovative method described in this paper offers several advantages. Firstly, the high SBR and real- time intraoperative imaging aid in precise and rapid identification of the target nerve tissue. Secondly, within the fluorescence spectra of this fluorophore, the background tissue displays minimal autofluorescence, reducing interference with nerve tissue identification. Lastly, this method is minimally invasive, enabling local or systemic administration without disrupting routine surgical procedures or adding additional surgical time. In this study, no adverse reactions were observed in experimental animals treated with Rhodamine B.

Certainly, limitations on the imaging depth may exist because the fluorescence spectra of Rhodamine B do not fall within the NIR range. But the image quality of visualization in OMI guided surgery is not composed of a single factor, so comprehensive consideration should be taken. The Ex/Em of Rhodamine B is 554 nm/576 nm, and the wavelength difference between excitation and radiation band is 20 nm, so the efficient separation of excitation light and radiation fluorescence can be completed by narrow bandwidth excitation light source and OD superior narrowband filter. In addition, by preparing development reagents with high fluorescence efficiency and developing image sensors with high quantum efficiency, and optimizing image saturation, image gain, image contrast and other parameters it is finally possible to obtain high SBR. And in experiments, we have got a lot of evidence for that. Over the past few decades, there have been significant advancements in OMI technology. The integration and improvement of this technology in conjunction with surgical procedures have successfully propelled the advancement of fields such as fluorescence-guided surgery, molecular imaging-guided surgery, and targeted surgery (60–66). This has paved the way for new possibilities in surgical advancement. This has paved the way for new directions in surgical advancement. The integration of this technology with nerve tissue imaging can effectively reduce intraoperative damage to surrounding nerves. By pushing the boundaries of surgical procedures, it holds the potential to bring about revolutionary breakthroughs in the field of surgery.

## 5 Conclusion

In this study, we successfully achieved fluorescence imaging of PNs using Rhodamine B in both animal experiments and fresh human nerve tissue. We explored the relationship between Rhodamine B dissolved in different formulations, administered at different doses, and the resulting SBR. Additionally, we investigated the metabolism and toxicity of Rhodamine B in mice through intravenous injection via the tail vein. The results of this research confirmed the clinical translational potential of Rhodamine B.

## Data Availability

The original contributions presented in the study are included in the article/Supplementary material, further inquiries can be directed to the corresponding authors.
